# Medical legislation – educational needs assessment for dental students

**Published:** 2017

**Authors:** SM Piţuru, A Nanu, A Bucur

**Affiliations:** * Discipline of Work Organization and Legislation in Dentistry, “Carol Davila” University of Medicine and Pharmacy, School of Dentistry, Bucharest, Romania; ** Oral and Maxillofacial Surgery Discipline, “Carol Davila” University of Medicine and Pharmacy, School of Dentistry, Bucharest, Romania

**Keywords:** medical legislation, educational needs, dental students

## Abstract

**Rationale (hypothesis)**: Many studies have highlighted the vulnerabilities in medical practice due to the legislation ignorance. Therefore, developing special programs for students training is needed and has become imperative.

**Objective**: This research aimed to identify the educational needs for the 5th year students in “Carol Davila” School of Dentistry in Bucharest, related to the legislation in dentistry and its area of application.

**Methods and results**: 199 students were invited to respond to a specially designed questionnaire. The questionnaire had 11 closed-response questions and the answers were statistically analyzed. The results indicated many educational needs in all the areas of investigation.

**Discussion**: “Carol Davila” University of Medicine and Pharmacy is the first university in Romania that created a new discipline in the School of Dentistry, called Work Organization and Legislation in Medicine and Dentistry.

## Introduction

Medical malpractice is defined as any error or omission by a physician/ dentist/ nurse/ pharmacist during any medical investigation or treatment of a patient, which is not consistent with the accepted norms of practice in that medical community and causes an injury to the patient. Moreover, malpractice may also be an ethical issue when it is directly linked to patient privacy, medical data or his informed consent [**1**-**3**].

Unfortunately, the medical practice in Romania does not respect the law every time [**4**]. Therefore, the doctor can easily be sued by the patients (malpractice) and the insurance policies will not be settled [**5**].

The Romanian healthcare legislation is new. The rules for implementing no. XVI Title “The civil liability of the medical personal and providers of medical and pharmaceuticals products and services” of law no. 95/ 2006 concerning the reform in health, modified and updated, were published in April 2007, the rest of the legislation being published since 2003.

The schools of dentistry training and postgraduate programs have no specific training in medical laws, patient’s rights, medical personal liability, or interaction with various healthcare institutions. Several key areas for medical laws standards, which are insufficiently known by the medical community, were identified in dentistry in Romania. 

The major aim of this study was to find the educational needs for the 5th year dental students, starting with their knowledge evaluation, related to some major key areas of medical law standards such as informed consent, medical emergencies protocols, privacy of the medical information, patients’ access to their own medical information, preventing overcoming medical competence. 

## Materials and Methods

A group of 199 students from “Carol Davila” School of Dentistry in Bucharest was invited to answer to a specially designed questionnaire. The sample of the 5th year dental students’ population was built in order to obtain a 95% confidence interval and a 3.98% estimated error by using the Google Sample Size Calculator. The questionnaire included 11 closed-response questions with multiple choices for the response. The questions and the right answers (bolded) are presented below:

“1. The dentist must prepare the following papers for the patient’s primary evidence: **a** – patient’s record; **b** – examinations’ records notebook; c – computer files. 

2. Can the details related to the patients’ dental treatment be shared?: a – yes, to NGOs who supported patients; b – yes, to the patients’ families members; **c** – no.

3. In case of a media event, is the press allowed to be present during the treatment?: **a** – yes, if the patient agrees; b – yes, the media must know; c – no.

4. Does the patient have access to his own dental record?: a – yes, he can take the original; **b** – yes, he can take a copy; c – no. 

5. Which are dental emergencies?: **a** – a dental pain; **b** – an oral infection; **c** – an oral bleeding.

6. Should the patients know the risks when a risky clinical procedure is about to be done? **a** – yes, the risks must always be explained in detail; **b** – no, because it is more important to protect the patient; c – no, because only the dentist should decide if the procedure must be done or not.

7. Is it always necessary to have the informed consent before the dental treatment?: a – no, the dental treatment is diced by the dentist only; b – yes, always; c – yes, only when major risks are involved.

8. Which is the right decision when a patient comes into a dental clinic and requires other health care?: a – the dentist is not allowed to perform other health care; b – the dentist may perform other health care after he consults a specialist of this kind; **c** – the dentist may perform other health care is case of an emergency.

9. Is it necessary for the dentist to know the dental clinic regulation policy?: **a** – yes; b – no; c – there is no such regulation policy in the dental units.

10. If the patient has a dental emergency and he is not able to give his consent, which is the document that the dentist needs?: a – the dentist does not need an informed consent in case of an emergency; b – a report written by a specialist; c – a decision from a special medical committee.

11. Do you think medical malpractice accusations represent an existing and real danger?: a – yes; b – no

## Results

**Fig. 1:**
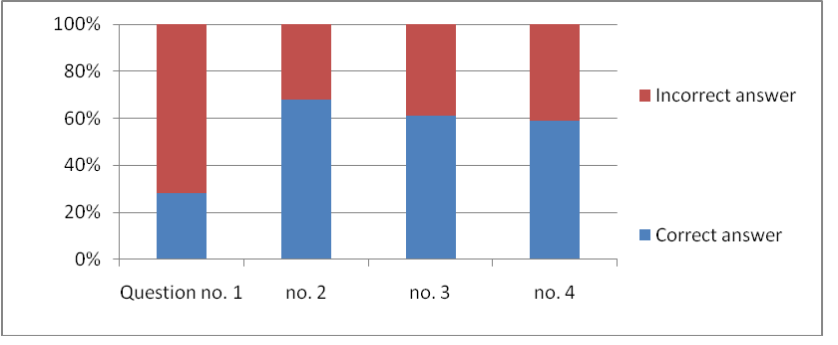
The responses for the 4 questions

**Fig. 2:**
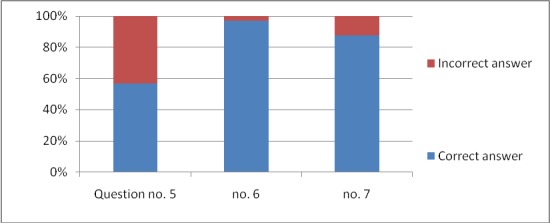
The responses for questions 5–7

**Fig. 3:**
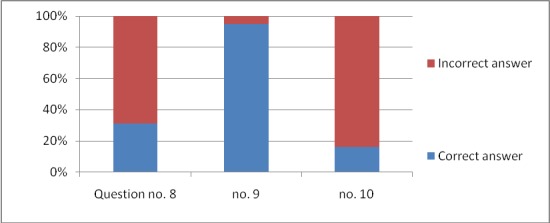
The responses for questions 8–10

**Fig. 4:**
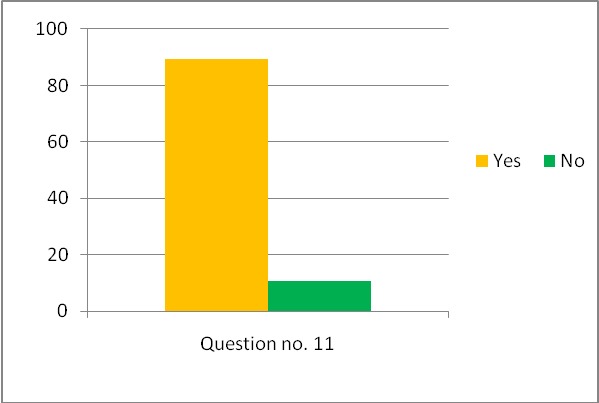
The responses for the last question (no. 11)

The responses to all the 11 questions are graphically expressed in **Fig. 1**–**4**.

## Discussion

None of the students involved in this study chose the right answers for all the questions. The 5th year students from “Carol Davila” University of Medicine and Pharmacy, School of Dentistry in Bucharest do not know the legal standards for the dental medical practice such as the informed consent, medical emergencies protocols, privacy of the medical information, and the access of patients to their own medical information, preventing overcoming medical competence.

The informed consent:

The informed consent is a very important element for the medical practice. The students’ educational needs related to this issue were investigated by using 3 questions (no. 6, 7 and 10). Most students agreed with the importance of the patients being informed prior to the obtaining of the informed consent. However, some of them did not know that the informed consent had to be signed by the patient and which the proper medical emergencies protocols were. 

Medical privacy

The analysis was focused on questions no. 2 and 3. Almost 60% of the students picked up the right answers for maintaining the medical privacy especially related to legal aspects.

The access of patients to their own medical information

41% of the students did not indicate right answers concerning the full access of patients to their own medical information. 

Preventing overcoming medical competence 

The dentist must be limited in dental practice by the competences he owns. It seems that almost 70% of the students involved in this study did not know the background and circumstances, which allowed the medical (dental) competence overcoming.

The last question did not test any knowledge and asked for the respondent’s personal opinion. 

Almost 90% of the students believed that dental malpractice is a real and current “danger” in dentistry. In the light of this, we believe that universities training programs must ensure the necessary information regarding the legal medical aspects, communication and interaction between the dentists and their patients, negotiation of dental malpractice insurance policy or interaction with various healthcare institutions, all these being “a must have” for an efficient and legal dental practice. 

The students in “Carol Davila” School of Dentistry in Bucharest received the right to perform dentistry right after their graduation. Not knowing or breaking the medical legislation can lead to the possibility that many dentists are sued by the patients. Moreover, the dental malpractice insurance policies are useless when the medical laws are broken. 

“Carol Davila” University and Pharmacy in Bucharest is the first university in Romania that has been training students in medical personnel liability and medical malpractice prevention and management starting with 2013 (optional courses and seminars for 5th year students). The training became mandatory starting with 2014 in “Carol Davila” University and Pharmacy, School of Dentistry in Bucharest (by Discipline of Work Organization and Legislation in Dentistry) and School of Nursing and Midwives (by Discipline of Medical Malpractice Prevention and Management).
